# An Innovative Use of Twitter to Disseminate and Promote Medical Student Scholarship During the COVID-19 Pandemic: Usability Study

**DOI:** 10.2196/33767

**Published:** 2022-07-13

**Authors:** Gary Allen, Jenna Garris, Luan Lawson, Timothy Reeder, Jennifer Crotty, Johanna Hannan, Kori Brewer

**Affiliations:** 1 Brody School of Medicine East Carolina University Greenville, NC United States; 2 Office of Medical Education Brody School of Medicine East Carolina University Greenville, NC United States; 3 Department of Emergency Medicine Brody School of Medicine East Carolina University Greenville, NC United States; 4 Department of Pediatrics Brody School of Medicine East Carolina University Greenville, NC United States; 5 Department of Physiology Brody School of Medicine East Carolina University Greenville, NC United States

**Keywords:** medical education, social media, web-based learning, innovation, Twitter, dissemination, scholarship, medical student, platform, academic promotion, COVID-19

## Abstract

**Background:**

Due to the emergence of the COVID-19 pandemic in March 2020, the cancellation of in-person learning activities forced every aspect of medical education and student engagement to pivot to a web-based format, including activities supporting the performance and dissemination of scholarly work. At that time, social media had been used to augment in-person conference learning, but it had not been used as the sole platform for scholarly abstract presentations.

**Objective:**

Our aim was to assess the feasibility of using Twitter to provide a completely web-based forum for real-time dissemination of and engagement with student scholarly work as an alternative to a traditional in-person poster presentation session.

**Methods:**

The Brody School of Medicine at East Carolina University launched an online Medical Student Scholarship Forum, using Twitter as a platform for students to present scholarly work and prepare for future web-based presentations. A single student forum participant created posts using a standardized template that incorporated student research descriptions, uniform promotional hashtags, and individual poster presentations. Tweets were released over 5 days and analytic data were collected from the Twitter platform. Outcome measures included impressions, engagements, retweets, likes, media engagements, and average daily engagement rate.

**Results:**

During the conference, the student leader published 63 tweets promoting the work of 58 students (55 medical and 3 dental students) over 5 days. During the forum and the following week, tweets from the @BrodyDistinctly Twitter account received 63,142 impressions and 7487 engagements, including 187 retweets, 1427 likes, and 2082 media engagements. During the 5 days of the forum, the average daily engagement rate was 12.72%.

**Conclusions:**

Using Twitter as a means of scholarly dissemination resulted in a larger viewing community compared to a traditional in-person event. Early evidence suggests that social media platforms may be an alternative to traditional scholarly presentations. Presenting via Twitter allowed students to receive instantaneous feedback and effectively network with wider academic communities. Additional research is needed to evaluate the effectiveness of knowledge uptake, feedback, and networking.

## Introduction

The emergence of COVID-19 created a period of intense uncertainty for both medical students and faculty. In the spring of 2020, cancellation of all in-person learning activities forced medical education and student engagement into a web-based format, including activities in support of performance and dissemination of medical student scholarship. Prior to the pandemic, educators at the Brody School of Medicine developed 4 longitudinal, paracurricular distinction tracks through which medical students could achieve recognition in research, service learning, medical education and teaching, or health system transformation and leadership [1]. Between the first and second year of medical school, students in each track enter an 8-week summer immersion experience with the opportunity to present their results locally to faculty and students at an annual Medical Student Scholarship Forum. COVID-19–related safety restrictions prevented traditional assembly of the forum, depriving scholars of the opportunity to create a poster or oral presentation, promote their work, experience professional interactions with peers and faculty, receive real-time feedback, and celebrate project success.

To address this problem, the distinction track leadership collaborated with East Carolina University news services to develop a student-driven web-based event, leveraging the advanced features of Twitter. Social media platforms, particularly Twitter, promote discussion of key clinical and medical education topics and disseminate evidence-based medicine [2,3]. A recent review highlighted the various educational opportunities available on Twitter, including the ability to engage in Twitter-based journal clubs, web-based case conferences, and “Tweetorials,” where a user, presumably an expert, explains an important topic or concept in a series of posts [2]. Further, many professional societies are “tweeting the meeting” at academic conferences to increase visibility and distribute content to a wider audience [3-6]. Limited but still successful, examples exist of web-based–only Twitter poster sessions, such as the annual #RSCPoster event held by the Royal College of Chemistry [7]. Our objective was to assess the feasibility of using Twitter to provide a completely web-based forum for real-time dissemination of and engagement with student scholarly work as an alternative to the traditional in-person Medical Student Scholarship Forum.

## Methods

### Study Population

Our study population included a total of 24 medical students participating in summer distinction track programs, as well as 32 medical and 3 dental students participating in the Summer Scholars Student Research Program, a precursor to the competitively selected Research Distinction Track. Medical and dental students not participating in the programs were excluded. Academically, all medical students were between their first and second year, whereas all dental students had yet to start their first-year curriculum.

### Event Preparation

Initially, we polled scholars regarding their preference for an indefinite postponement of this in-person event or the development of a web-based event. Scholars overwhelmingly preferred the opportunity to present their projects using a web-based approach; a Twitter account (@BrodyDistinctly) already existed to promote the activities of Distinction Track Scholars. Distinction track leadership collaborated with a representative student scholar to design a web-based event using Twitter as the platform. Prior to the event, East Carolina University’s news services office provided education regarding professional “best practices” for the use of Twitter [8]. Additionally, scholars attended an educational session on the responsible use of Twitter and were encouraged to set up a professional Twitter account.

**Figure 1:**
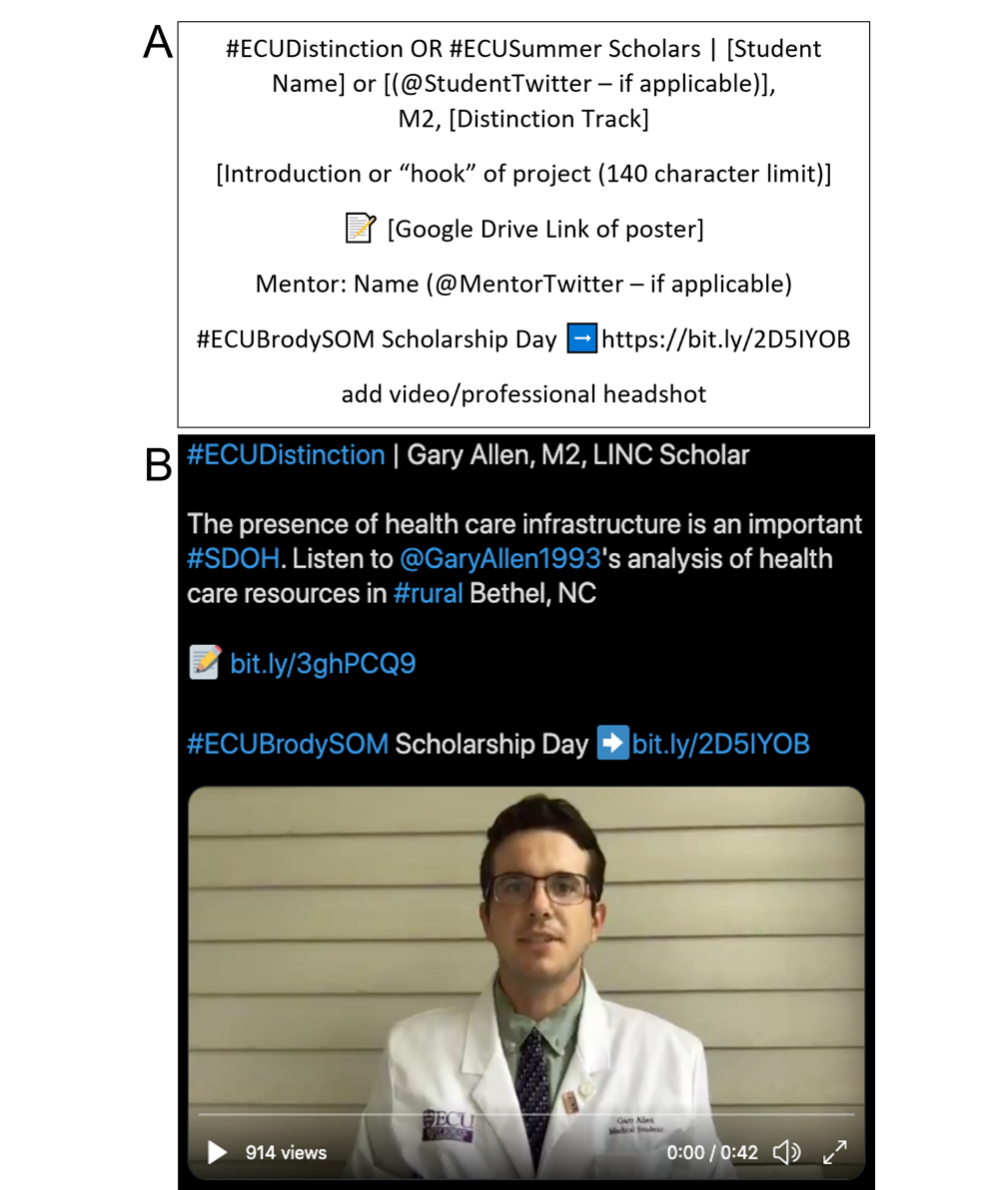
(A) Sample template with guidelines provided to students to compose their tweet; (B) example of a finalized tweet that was posted for the online Medical Student Scholarship Forum event on Twitter.

To facilitate creation of posts, we developed a standard template that included all desired elements (Figure 1). Standard template elements included the student’s preferred name, academic year, and distinction track, as well as specific university-approved hashtags used to promote the web-based event. As a final assignment, each scholar submitted additional elements needed to create their own individual post, using the template. These sections included a “hook” or headline for their individual project and their final poster, using a university-approved template. Students were then given the option to either submit a 1-minute video to describe the results and significance of their work or allow track leadership to use their professional headshot. Web links to the poster presentations stored on a file sharing service were included in each post. Finally, the standard template included the names of the research mentors and a link to a university webpage highlighting the event.

Of note, Twitter limits posts to 280 characters, so inclusion of all desired elements required innovative solutions. First, web links to poster presentations were shortened using a free URL shortening service. Additionally, electronic ideograms, commonly referred to as emojis, were used to replace words wherever possible. Lastly, students were asked to limit their research introductions to less than 140 characters. On occasion, students were asked to further shorten “hooks” to account for variations in name length among students and mentors.

Once all materials were received, the student scholar representative used TweetDeck, a free application provided by Twitter Inc, to schedule 12 posts each day, separated by 1 hour, for 5 consecutive days, from participating scholars. All content was distributed from the @BrodyDistinctly Twitter account. The distinction track leaders created this account in April 2019 to promote Distinction Track Scholars’ activities.

The event was advertised through the health sciences campuswide email listserv (with 3292 recipients) and tweeted from the Brody School of Medicine (@ECUBrodySOM; 2936 followers) and Distinction Track (@BrodyDistinctly; 172 followers) Twitter accounts. The week preceding the event, details regarding the schedule of the individual presentations were posted to the Distinction Track Twitter feed.

### Outcome Measures

Our main outcome measures were impressions (ie, the number of times people viewed a post on Twitter), (2) engagements (ie, the number of times people interacted with a post), (3) likes (ie, the number of times people liked a post), (4) detail expands (ie, the number of times people viewed the details of a post), and (5) retweets (ie, the number of times people retweeted a post) [9]. Lastly, we also tracked the engagement rate (ie, the number of engagements divided by the number of impressions), a common metric used by both professionals and academics to evaluate the overall performance of tweets [10,11]. These outcomes are commonly used in professional societies as measures of successful dissemination of meeting content [11,12].

### Analysis Methods

All data were obtained for free, using Twitter’s basic analytics services available to all users. Date filters were used to collect data within the time frame of one week before, during, and one week after the event. Of note, analysis of the types of users (ie, students, local faculty, or outside faculty) who engaged with posts was not available for free on the Twitter platform.

### Ethical Considerations

The current study was deemed not to constitute human research using the Human Research Determination Worksheet provided by the University and Medical Center Institutional Review Board of East Carolina University. This decision was confirmed by the IRB and no ethical approvals were sought.

## Results

### Principal Findings

**Figure 2:**
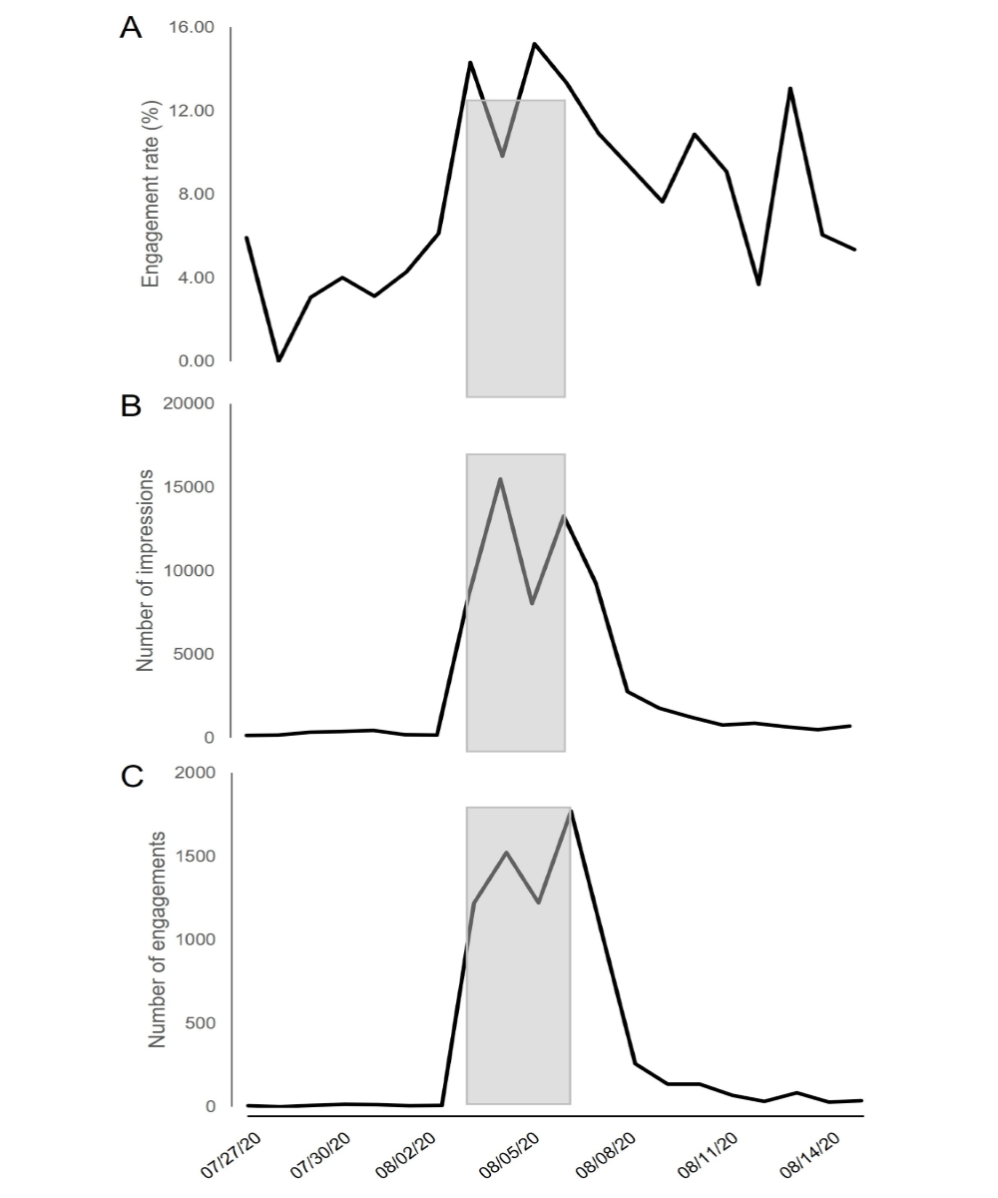
Twitter analytics of the (A) engagement rate, (B) number of impressions, and (C) engagements with the @BrodyDistinctly account before (July 27-August 2, 2020), during (August 3-7, 2020), and after (August 8-14, 2020) the web-based Medical Student Scholarship Forum. The gray shaded area represents the 5 days of the web-based event.

The web-based Medical Student Scholarship Forum took place over 5 days (August 3-7, 2020). Data are summarized in Figure 2. In the week leading up to the forum (July 27-August 2, 2020), the daily engagement rate was 3.43% (57/1662). During the forum, a total of 63 tweets promoted the work of 58 student scholars (55 medical and 3 dental students). Throughout the forum and the week following it (August 3-14, 2020), tweets from the @BrodyDistinctly Twitter account received 63,142 impressions, 7487 engagements, 1427 likes, 2082 detail expands, and 187 retweets. During the 5 days of the forum, the average daily engagement rate was 12.72% (6743/54,588)—an increase of roughly 270% from the week prior to the event. Engagement continued the week following the forum despite no additional tweets from the @BrodyDistinctly account. The postforum average daily engagement rate was 8.53% (744/8554). During or after the forum, the highest daily engagement rate was 15.2% (1221/8025), and the lowest daily engagement rate was 3.6% (32/869). The top tweet (ie, the tweet receiving the highest number of impressions) of the event—“Drink intake is higher from 100% juice and juice-flavored drinks compared to soda and sweet tea in children with severe obesity”—earned 5855 impressions. The top media tweet (ie, the tweet with photo, video, or Vine that received the highest number of impressions)—“Outreach in diabetic and hypertensive patients serves to educate and provide necessary resources during the #COVID19 #pandemic”—earned 2705 impressions. Additionally, there were a total of 14 comments, 11 of which were generally positive (eg, “good job!”), and 3 were irrelevant. In August, the @BrodyDistinctly Twitter account acquired 49 new followers and had 2032 profile visits. Only 4 additional tweets were published from the @BrodyDistinctly account during the month of August. Prior in-person Medical Student Scholarship Forums have included approximately 65 student poster presentations and an estimated 70 faculty and student attendees. Strict in-person counts of nonpresenting attendees have not been recorded in previous years.

A total of 5 medical students from all distinction tracks were individually asked to identify the strengths and weaknesses of the web-based–only forum. They cited early exposure to web-based medical communities, convenience to create materials and participate in the conference, and distribution of their work to a wider audience as strengths of the web-based–only conference. Additionally, many recognized the importance of understanding how to create posts to promote their work in the future. Weaknesses identified included lack of prolonged discussions, critical feedback, and questions regarding their work. Additionally, some students lacked a Twitter account and chose not to make one for the event. Lastly, the design of the tweets, with research “hooks” in the tweet and posters being embedded as hyperlinks, may have decreased the total number of posters thoroughly reviewed by each participant.

## Discussion

In response to unanticipated limitations for in-person gatherings due to the COVID-19 pandemic, our Twitter-based celebration of scholarship served as a successful substitute to a traditional in-person poster presentation session. Our results show that the event allowed dissemination of student scholarship to a wider audience than previously possible. Prior in-person Medical Student Scholarship Forums have included around 65 student poster presentations and approximately 70 faculty and student attendees. During the web-based forum, the number of engagements (7487) exceeded the number of faculty and students at the School of Medicine (approximately 343 students and 445 full-time faculty). Additionally, analysis indicates that the content of student posts, which included our standard template, a research “hook,” and various hashtags, was crafted in a way to capture the attention of users who viewed them, otherwise referred to as “engagement rate.” Multiple sources report that a “good” Twitter engagement rate ranges from 0.2% to 0.9%, with the August 2020 rate reported to be 0.18% [13]. In academia, studies have suggested that a rate of roughly 7% is considered “high engagement” [12]. Our average daily engagement rate during the 5 days of the web-based forum was 12.72%, and the average daily rate remained elevated at 8.53% for 7 days afterward. Furthermore, our report may still underestimate the overall scope of impressions and engagements due to the variability in the ways that students promoted themselves. Most notably “quoted retweets,” in which the tweet is reposted by someone with an added comment of their own, are not included in the engagement or impressions analytics of the @BrodyDistinctly Twitter account. Lastly, scholars learned how to use social media for professional self-promotion and engagement [14].

The combination of our initial survey, where students chose to have a web-based–only scholarship forum rather than no event, and focus group themes indicated general student satisfaction with the event. Students cited early introduction to #MedTwitter, a popular Twitter thread for medical professionals, as well as practicing promoting themselves on social media as advantages to the web-based event. Disadvantages mentioned included less critical feedback and interaction with poster presentations than would have been likely in an in-person poster session.

A growing body of evidence describes the incorporation of concurrent Twitter use into in-person academic conferences, and examples exist of successful web-based–only academic poster presentations [3-6,9]. Our analysis suggests that an entirely web-based forum displaying the scholar’s projects can be an effective means to disseminate the outcomes of summer immersion programs and promote scholarly work. Considering the evidence base and the advantages identified in this analysis, we plan to continue to use Twitter to advertise and complement future in-person and web-based events. Moving forward, we will institute a dated, event-specific hashtag that can be updated annually (ie, #BSOMScholars21). We will also work to grow our Twitter followers so that the scholars’ presentations can reach a wider audience. As others seek to implement novel strategies to disseminate knowledge and network, development of best practices and approaches for social media use are necessary to effectively harness its educational and professional development potential. Future research will need to examine the relationship of reader engagement with learning and determine whether student participation in such events enhances their visibility within residency programs of interest.

Limitations of our analysis include inability to identify demographic trends of individuals who interacted with the posts. For instance, the current analytics cannot differentiate the interaction of medical school faculty who can provide career opportunities from the interaction of a supportive family member or one of the authors. Additionally, although the @BrodyDistinctly Twitter account gained 49 new followers in August, many of these were likely students who created a profile to participate in the event. Finally, as quoted retweets and any activity they generate are not measured in our program analytics, we may be underestimating the reach of our Twitter forum.
